# Gonadal Hormone Influences on Sex Differences in Binge Eating Across Development

**DOI:** 10.1007/s11920-021-01287-z

**Published:** 2021-10-06

**Authors:** Megan E. Mikhail, Carolina Anaya, Kristen M. Culbert, Cheryl L. Sisk, Alexander Johnson, Kelly L. Klump

**Affiliations:** 1 Department of Psychology, Michigan State University, 316 Physics Rd., Room 107B, East Lansing, MI, 48824-1116, USA; 2 Department of Family Medicine and Public Health Sciences, Wayne State University School of Medicine, Detroit, MI, USA; 3 Neuroscience Program, Michigan State University, East Lansing, MI, USA

**Keywords:** Binge eating, Estradiol, Progesterone, Testosterone, Puberty, Sex differences

## Abstract

**Purpose of Review:**

Binge eating is a transdiagnostic symptom that disproportionately affects females. Sexually dimorphic gonadal hormones (e.g., estradiol, testosterone) substantially impact eating behavior and may contribute to sex differences in binge eating. We examine recent evidence for the role of gonadal hormones in binge eating risk across development.

**Recent Findings:**

Both organizational (long-lasting impact on the central nervous system (CNS)) and activational (transient influences on the CNS) hormone effects may contribute to sex differences in binge eating. Gonadal hormones also impact within-sex variability in binge eating, with higher estradiol levels in females and higher testosterone levels in males protective across development. Emerging evidence suggests that the impact of gonadal hormones may be greatest for people with other risk factors, including genetic, temperamental (e.g., high negative affect), and psychosocial (e.g., exposure to weight-based teasing) risk.

**Summary:**

Gonadal hormones contribute to sex differences and within-sex variability in binge eating across development.

## Introduction

Binge eating (i.e., eating large amounts of food, accompanied by loss of control) is a core, transdiagnostic eating disorder (ED) symptom associated with considerable distress [1–3], impairment [4,5], and medical/psychiatric comorbidity [6,7]. Binge eating is also common, affecting approximately 10% of women, and a smaller but significant number of men [8–10]. The economic costs of binge eating and related EDs (including bulimia nervosa [BN], binge-eating disorder [BED], and the binge eating/purging subtype of anorexia nervosa [AN-BP]) are substantial [11]. While existing treatments can decrease the frequency of binge eating, more than half of people continue to experience binge eating even after treatment [12,13]. Better understanding of the factors that lead to binge eating is important to advance research and intervention.

The epidemiology and typical developmental trajectory of binge eating provide insight into its etiology. Notably, binge eating and related behaviors are more common in females than males among both humans [8,14–16] and animals [17,18]. The prevalence of binge eating and related EDs also increases dramatically during puberty, with substantially greater increases experienced by girls [14,19] and female animals [18,20]. Though sex differences in rates of binge eating are shaped in part by sociocultural influences (e.g., greater pressure to diet among girls [21]), robust replication of sex differences that increase at puberty across both culturally diverse human populations [22–25] and animals suggests an important role for sex-specific biological factors. Gonadal hormones (i.e., estradiol, progesterone, testosterone) may be particularly critical, as levels of these hormones are sexually dimorphic, impact eating behavior [26–28], and act on neural systems, such as serotonergic and dopaminergic circuits, believed to be dysregulated in EDs [29–31].

Gonadal hormone effects underlie sexual differentiation of the brain and behavior, and may be organizational or activational in nature [32]. Organizational effects permanently shape the structure and later function of neural systems without necessarily immediately impacting behavior [33,34]. The prenatal/perinatal period and puberty are both sensitive periods for organizational effects of gonadal hormones [35,36], permanently priming the brain to respond to hormones in a sex-dependent manner later in development. Conversely, activational effects immediately and transiently affect neural signaling and behavior and are most salient in adulthood. Normative activational effects often depend on earlier hormonal organization [33,34].

**Figure 1:**
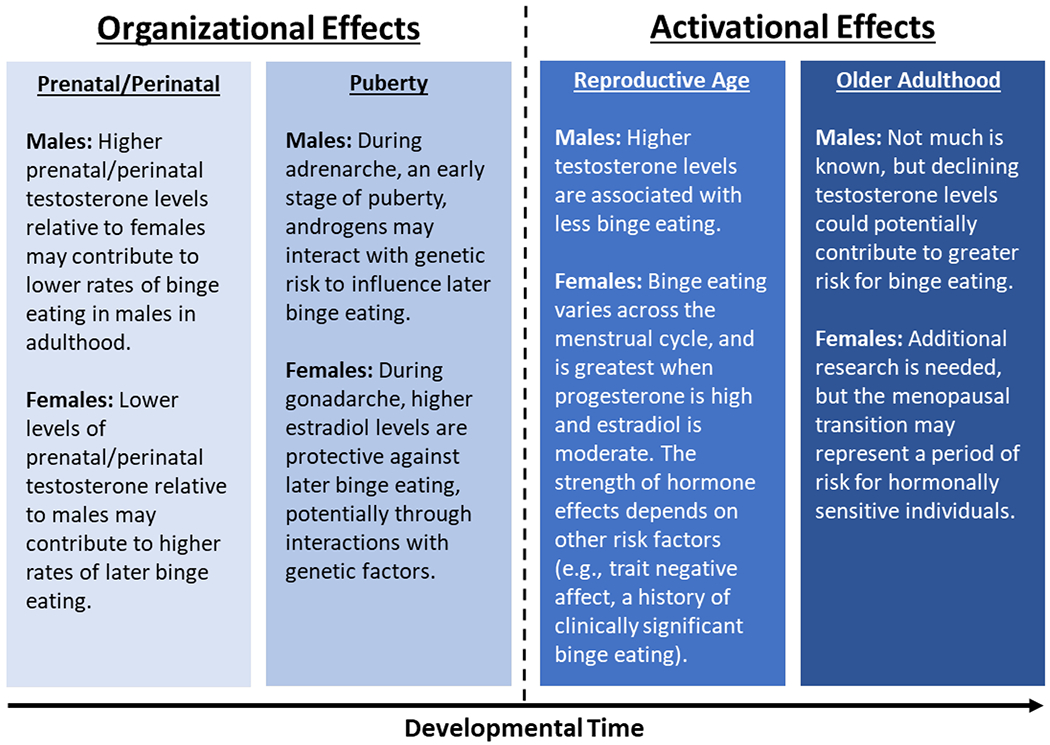
A summary of hormone effects on binge eating across development in females and males

In this review, we will examine evidence for organizational and activational gonadal hormone effects on binge eating risk across development, with a focus on research published in the past 5 years. We will focus on four key reproductive stages: the prenatal/perinatal period, puberty, adult reproductive age, and midlife, including the menopausal transition (see Figure 1]. For each stage, we will discuss the impact of gonadal hormones on binge eating risk, including hormonal contributions to sex differences and within-sex variability in binge eating. Though our review will focus on binge eating, we will also discuss closely related forms of dysregulated eating, such as emotional eating (i.e., eating in response to negative feelings). Both human and animal studies will be included to leverage the unique capability of animal studies to disentangle biological from sociocultural influences.

## The Prenatal/Perinatal Period: a Key Stage for Brain Organization

Gonadal hormones balance drives for eating and mating to maximize survival and reproductive success in adulthood [37]. and the ability of the adult brain to respond to these signals is organized by hormones prior to and shortly after birth. Correspondingly, greater prenatal/perinatal testosterone exposure in males relative to females predicts later sex differences in eating behavior, including greater food intake and reduced preference for sweet foods among males [28,38, 39]. Accumulating evidence suggests that prenatal/perinatal testosterone may also impact later susceptibility to dysregulated eating.

Recent experiments in animals indicate that prenatal/perinatal testosterone has a marked impact on binge eating in adulthood. In rodent models, animals are identified as “binge eating prone” (BEP) if they consistently consume high quantities of palatable food (e.g., frosting) when given brief, intermittent access. Ordinarily, female rats show 2–6× higher rates of BEP phenotypes than males in adulthood [17]. However, Culbert et al. [18] found that these sex differences could be largely eliminated by treating female rats with testosterone immediately after birth. Strikingly, female rats exposed to a male-like perinatal hormonal milieu through exogenously administered testosterone were significantly less likely than untreated females to be BEP in adulthood, and were in fact no more likely to be BEP than males [18]. While the phenomenology and likely etiology of binge eating are considerably more complex in humans, these findings suggest that sex differences in rates of binge eating in adulthood are driven, in part, by differential brain organization by hormones early in life.

It is more difficult to study the impact of prenatal/perinatal hormones on later eating behavior in humans because we cannot directly manipulate participants’ testosterone levels. Instead, research in humans has focused on how individual differences in indirect measures of prenatal testosterone correlate with binge eating in adolescence and adulthood. Two primary measures of prenatal testosterone have been examined. First, females gestated with a male cotwin are thought to be exposed to higher testosterone levels in utero than other females [40]. Females with a male cotwin would therefore be expected to experience less binge eating than females without a male cotwin. Alternatively, researchers have examined the ratio of the length of the index finger to the length of the ring finger (i.e., 2D:4D ratio), with lower 2D:4D ratios thought to reflect greater prenatal testosterone exposure [41,42].

Results from studies using these indirect measures of prenatal testosterone have been inconsistent, with some showing lower rates of binge eating and BN among people thought to be exposed to greater prenatal testosterone [43,44], some showing no differences [45–47], and one study showing the opposite association [48]. Studies of disordered eating symptoms more broadly have been similarly mixed [47,49–52]. While most studies have only examined women, Kothari et al. [48] found no association between 2D:4D ratio and risk for BN among men.

Several explanations for inconsistent findings in humans are possible. Elevated prenatal/perinatal testosterone could have the strongest impact on later binge eating when testosterone is also relatively high in adolescence/adulthood [53,54], as typically occurs for men, but not women. However, the Culbert et al. [18] study discussed above observed protective effects of perinatal testosterone on BEP phenotypes in female rats even without administration of testosterone in adolescence/adulthood, suggesting that the protective effects of testosterone early in life do not fully depend on later testosterone exposure. Alternatively, it may be that gestation with a male cotwin or other naturally occurring variations in prenatal/perinatal testosterone among girls do not provide enough testosterone to fully protect against later binge eating. Consistent with this possibility, female rats treated with testosterone in Culbert et al. [18] were considerably more masculinized (e.g., they showed altered pubertal development) than is observed in women with a male cotwin. Large mean sex differences in prenatal/perinatal testosterone exposure may therefore be more consequential for later binge eating than comparatively modest within-sex variability in prenatal/perinatal testosterone. A third possibility is that the effects of modestly elevated prenatal/perinatal testosterone on later dysregulated eating may depend on other factors, such as the presence of genetic or environmental risk. For example, elevations in prenatal/perinatal testosterone (e.g., from a male cotwin) may protect against binge eating in low-risk environments, but have little effect in the presence of substantial environmental stressors (e.g., pressures to diet or intense psychological stress). Evidence in favor of this latter hypothesis comes from research showing that the association between prenatal testosterone and disordered eating in girls is weaker during developmental periods associated with increased overall ED risk [55].

In aggregate, research suggests that sex differences in binge eating may be traced in part to differential prenatal/perinatal testosterone exposure, with higher testosterone among males protecting against later dysregulated eating. Additional research is needed to more conclusively determine the extent to which within-sex variability in prenatal/perinatal testosterone may impact risk, and whether higher doses of testosterone (e.g., as experienced by girls with congenital adrenal hyperplasia [56]) may have a stronger influence on binge eating in girls. Other promising avenues for research include further investigation of developmental shifts in prenatal/perinatal testosterone influences, and possible interactions between early testosterone exposure and psychosocial risk factors.

## Puberty: Organization, Redux

Puberty has emerged in recent decades as a crucial second sensitive period for shaping adult neural pathways [36,57]. Puberty can be divided into two distinct but overlapping phases. Adrenarche is an early stage of puberty defined by rising adrenal androgen levels (i.e., dehydroepiandrosterone, dehydroepiandrosterone-sulfate, androstenedione) that is initially accompanied by few observable physical changes [58]. Gonadarche typically begins after adrenarche is underway, and involves increases in ovarian (in females) or testicular (in males) hormones that contribute to secondary sex characteristics, such as breast growth and voice deepening [58]. Both adrenarche and gonadarche appear important in determining later binge eating risk, but their effects differ somewhat across sex. Specifically, the increase in adrenal androgens at adrenarche appears to be more consequential for organizing later binge eating in boys, while the increase in estradiol at gonadarche plays a critical role in girls. Notably, progesterone does not increase significantly until late puberty, after menarche, and thus is thought to be less influential in pubertal changes in binge eating in girls [58]. Sex differences in responsivity to hormones at puberty may reflect earlier differences in prenatal/perinatal testosterone exposure and corresponding brain organization, which “prime” males to respond to androgens, and females to estrogens, later in development [36].

In males, the increase in androgens at adrenarche overlaps with increased expression of latent genetic individual differences in ED risk. Prior to adrenarche, there are few detectable genetic influences on ED symptoms (including binge eating) in both boys and girls [59–61]. However, during adrenarche, the heritability of disordered eating increases to over 50% in boys (similar to heritability in adulthood) [59]. This increase in heritability may be driven by hormonally regulated changes in gene expression, whereby androgens upregulate or downregulate transcription of genes relevant to disordered eating [62]. These changes in gene expression may in turn shape brain development in a way that leaves a lasting impact on binge eating risk in adulthood. The increase in heritability of disordered eating during adrenarche in boys occurs without a corresponding increase in phenotypic ED symptoms [59], consistent with organizational rather than activational effects of androgens at this time. Unlike boys, girls do not show an increase in the heritability of disordered eating during adrenarche [59], potentially due to lower responsiveness to androgens in girls following lower prenatal/perinatal testosterone exposure.

Research on adrenarche is still nascent and has so far relied on indirect measures to determine adrenarche status (e.g., age and outward developmental signs). Additional studies that directly measure adrenal androgen levels are needed to more fully understand the impact of adrenarche on binge eating at genetic and phenotypic levels. It is also unclear how within-sex differences in androgen levels at adrenarche may impact disordered eating, and if higher androgen levels may facilitate the expression of protective genes, or inhibit the expression of “risky” genes, in boys.

By early gonadarche, genetic influences on disordered eating appear fully online in boys [59,60]. Androgens continue to influence risk for disordered eating during later stages of puberty in boys, but effects are more activational in nature, with negative phenotypic correlations between testosterone levels and disordered eating [54]. In contrast, ovarian hormones show minimal phenotypic associations with disordered eating during gonadarche in girls, but genetic influences on binge eating and other disordered eating symptoms increase precipitously, from ~0% in pre/early puberty to ~50% in mid/late puberty [60,61,63–66]. Gonadarche in girls may therefore parallel adrenarche in boys as a key organizational period during which ovarian hormones interact with genetic individual differences to influence later binge eating.

While genetic influences on binge eating and disordered eating increase across gonadarche in girls on average, not all girls experience this increase to the same degree. Because sex differences in binge eating are greater post-puberty [14,18,19], one might intuitively expect that girls with higher estradiol levels at puberty would experience greater increases in genetic influences on disordered eating. However, recent research has found that girls with higher estradiol levels during gonadarche show *weaker* genetic influences on binge eating than girls with relatively low estradiol levels [67]. Thus, it may be that higher estradiol levels inhibit expression of genes that increase risk for binge eating, and the increase in genetic influences across gonadarche in girls is driven primarily by girls with relatively low estradiol. Within-sex variability in estradiol appears to have primarily organizational, rather than activational, implications during gonadarche, as estradiol levels are not correlated with phenotypic binge eating symptoms at this stage [67].

Results of experimental animal studies are consistent with the hypothesis that low pubertal estradiol increases risk for later binge eating in females. Specifically, rates of BEP phenotypes [68] and palatable food consumption [69] in adulthood are significantly higher in female rats that have undergone pre-pubertal ovariectomy, which removes the body’s primary source of estradiol. These effects on binge eating only become apparent in late adolescence [68,69], consistent with an organizational (rather activational) impact of estradiol during puberty. Interestingly, the effects of pre-pubertal ovariectomy on palatable food consumption are greatest for BEP rats [68]. This result is consistent with a possible hormone x gene interaction, in which low pubertal estradiol may amplify a genetic predisposition for binge eating to produce more extreme BEP phenotypes in females with preexisting genetic risk. It is also consistent with human research showing that genetic individual differences have a more pronounced impact on binge eating when pubertal estradiol is low [67].

Several questions regarding the effects of pubertal gonadal hormones on binge eating remain. Longitudinal studies are needed to confirm that puberty is indeed a sensitive period that permanently and irreversibly organizes binge eating risk in a way that is distinct from the activational effects of hormones in adulthood. Additionally, the precise genes and neural pathways impacted by pubertal hormones are not yet known. Recent research showing that organizational estradiol effects are unique to palatable food rather than all food consumption [69] suggests a role for reward-related circuitry (e.g., dopamine pathways), but additional work is needed to better understand the mechanisms of pubertal hormone effects and their lasting contributions to sex differences in binge eating.

## Reproductive Age: Dynamic Activational Hormone Effects

By adulthood, organization of neural circuits by gonadal hormones appears mostly complete, and effects are more activational in nature [32]. Androgens continue to be protective in males, with higher testosterone levels associated with less binge eating in young adult men [53]. Interestingly, testosterone may have the opposite effect in adult women [70]. For example, women with polycystic ovary syndrome, a reproductive disorder characterized by elevated testosterone levels, have increased rates of BN and BED [71]. The few intervention studies that have actively manipulated testosterone levels in women with threshold EDs also suggest that elevated testosterone fails to improve binge eating [72], and may even increase risk [73]. Adult sex differences in the impact of testosterone on binge eating may reflect sex differences in exposure to androgens in early development. Without exposure to high androgen levels in utero, the female brain may respond differently to testosterone in adulthood, leading to positive (rather than negative) associations between testosterone and binge eating.

Evidence for the impact of ovarian hormones on binge eating in adult females comes from both ovariectomy studies in animals, and studies that have examined natural fluctuations in hormones across the menstrual cycle in women. Adult ovariectomy increases palatable food consumption in female rats [74], but does not change classification of rats as relatively BEP or binge eating resistant [68,74], suggesting protective activational, but not organizational, effects of ovarian hormones in adulthood. The effects of adult ovariectomy on binge-like behavior in female rats are particularly pronounced in the presence of other conditions that mimic risk factors for binge eating in humans, such as food restriction concurrent with seeing and smelling palatable foods [72]. Though ovariectomy removes both estradiol and progesterone, replacement with estradiol alone is sufficient to reverse the increase in binge-like behavior accompanying ovariectomy in rats [75], suggesting that estradiol is primarily responsible for the protective activational effects of ovarian hormones on binge eating in adult females.

**Figure 2:**
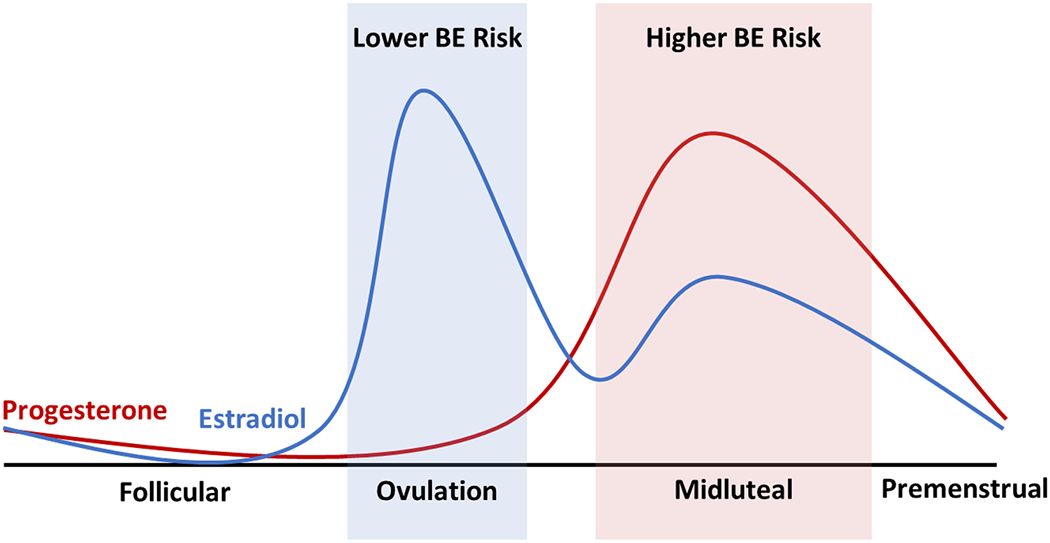
Changes in ovarian hormone levels and risk for binge eating (BE) across the menstrual cycle. Ovarian hormone levels and risk for binge eating change in predictable patterns across the menstrual cycle in women. Risk for binge eating and emotional eating is lowest during ovulation, when estradiol peaks and progesterone is low, and greatest during the midluteal phase, when progesterone peaks and estradiol is moderate

While it is generally not possible to experimentally manipulate ovarian hormones in women, changes in estradiol and progesterone across the menstrual cycle provide a natural experiment regarding ovarian hormone effects. Several studies have found that binge eating and emotional eating are lowest near ovulation, when estradiol peaks, and greatest in the mid-luteal phase, when progesterone is high and estradiol is moderate [77–79] (see Figure 2]. When ovarian hormone levels are examined directly, the interaction between estradiol and progesterone is the strongest predictor of dysregulated eating, which peaks when estradiol and progesterone are both high [77,80]. Progesterone is known to counteract the effects of estradiol [81]. The estradiol x progesterone interaction observed in these studies therefore likely reflects inhibition of the protective effects of estradiol on binge eating by progesterone. Interestingly, the magnitude of change in hormone levels from before to after ovulation appears less influential than the level of each hormone on a given day [82].

Though ovarian hormone influences on dysregulated eating are evident in both clinical and community samples, effects are stronger in women with clinically significant binge eating [83], a diagnosis of BN [79], or persistently high negative affect [84], suggesting possible interactions with other risk factors. Genetics may play a role in individual differences in risk, as heritability of dysregulated eating as estimated through twin designs is greatest during the midluteal phase of the menstrual cycle [85]. One hypothesis is that estradiol may inhibit expression of risky genes in vulnerable women during the first half of the cycle, and progesterone may amplify expression of these genes indirectly by inhibiting estradiol during the second half of the cycle. This hypothesis is consistent with pubertal findings suggesting reduced expression of genes impacting individual differences in binge eating when estradiol is high [67]. Genes influenced by changing hormonal milieus across the cycle could code for proteins involved in serotonergic or dopaminergic neural circuits [86,87] that are implicated in affective processes and binge eating [88]. Social/environmental stressors may also influence the strength of ovarian hormone-binge eating associations [89]. Indeed, research has demonstrated that associations between ovarian hormones and other ED symptoms may differ according to the environmental context. For example, Forney and colleagues [90] found that progesterone levels were associated with body image concerns in girls, but only in the presence of other psychosocial stressors (e.g., weight-related teasing).

In summary, gonadal hormones continue to play an important role in binge eating risk in adulthood in both males and females, but hormone effects shift from being organizational to activational in nature. As during puberty, the types of hormones that most strongly impact binge eating differ across sex, with testosterone being protective in males, and estradiol protective in females. During phases of the menstrual cycle when progesterone is high, women with genetic/psychosocial vulnerabilities experience a cyclical increase in binge eating that is not observed for men, which may contribute to sex differences in risk.

## Midlife: a Second Critical Transition?

Though most research to date has focused on younger adults, the substantial hormonal and psychosocial changes that occur in mid and later adulthood may also impact risk for binge eating. This may be particularly true for women approaching the menopausal transition, when reductions in protective estradiol levels could potentially lead to an intensification or relapse of binge eating [91].

The few community-based studies that have examined changes in disordered eating accompanying the menopausal transition have not consistently found mean differences in binge eating or disordered eating across menopause, with one study finding increased disordered eating during perimenopause [92], and two others finding no differences across menopausal status [93,94]. However, these studies have some significant methodological limitations, including cross-sectional (rather than longitudinal) designs and no direct measures of ovarian hormones. There is also some evidence that menopausal status may affect disordered eating more strongly for women with other risk factors, such as high levels of body comparison [95]. In addition, Mangweth-Matzek and colleagues [94] found that hormone-related menopausal symptoms (e.g., hot flashes) were more strongly associated with disordered eating than menopausal status per se, which could suggest stronger associations between menopausal hormonal changes and disordered eating for people who are more hormonally sensitive (who may be at higher risk for binge eating in general [96]).

Further research is needed to identify the people at greatest risk for increases in dysregulated eating across the menopausal transition, and the role of prior ED symptomatology, genetic/biological factors, and psychosocial factors in contributing to individual differences in risk. More research is also needed regarding hormonal shifts in males in mid-to-late adulthood. While midlife hormonal changes are more subtle in men, one recent study found that men ages 40–75 who endorsed more symptoms that could indicate lower testosterone (e.g., tiredness, reduced libido) reported greater disordered eating [97]. Longitudinal studies of testosterone levels in older men could help illuminate how aging-related hormonal changes may impact binge eating in males, perhaps contributing to reductions in sex differences in disordered eating observed in some studies later in life [98].

## Conclusion

Evidence across species and culturally diverse human populations consistently indicates a role for gonadal hormones in sex differences and developmental changes in binge eating. During the prenatal/perinatal period, higher testosterone levels in males appear to organize the brain in a manner that decreases binge eating risk in adulthood [18]. While prenatal/perinatal testosterone may critically influence sex differences in binge eating, the effects of within-sex variability in testosterone on later disordered eating are less clear, and may depend on the presence of additional environmental or psychosocial risk [55]. Boys experience increased expression of individual differences in genetic risk for disordered eating during adrenarche [59], perhaps reflecting a second period of brain organization that is shaped by androgen-regulated gene expression. Among girls, the onset of gonadarche is similarly associated with increased expression of genetic influences on binge eating [51,61]. Interestingly, higher estrogen levels during gonadarche appear protective in females across species. Pre-pubertal ovariectomy leads to substantial increases in BEP phenotypes in adulthood in female rats [68], and lower estradiol levels in girls during puberty are associated with greater genetic influences on binge eating (potentially reflecting increased expression of “risky genes”) [67]. Testosterone (in males) and estradiol (in females) continue to be protective against binge eating in adulthood [77,79]. Relatively little research has examined the influence of hormones later in life, particularly during the menopausal transition. However, preliminary evidence suggests that people who are more sensitive to hormonal changes may experience an increase in disordered eating at this time [94]. The association between midlife hormonal changes and binge eating is an important area for future research, especially given established associations between the menopausal transition and other phenotypes closely related to binge eating (e.g., depression [99]).

Findings to date have implications for both research and clinical practice. From a clinical perspective, educating clients on the role of biological factors (including hormones) on risk for binge eating may help reduce the self-blame and stigma that often accompany EDs [100]. It may also be helpful to discuss the impact of hormonal changes across the menstrual cycle on eating, and to work with clients who menstruate to track changes in ED symptoms across their cycle and plan ahead with strategies for coping with binge eating urges during risky hormonal milieus. With respect to research, an understanding of sex differences and developmental shifts in binge eating is incomplete without consideration of gonadal hormones and other biological factors that differ across sex and change across time. Careful research designs are needed to disentangle the unique contributions of biological and sociocultural factors when examining sex differences and developmental changes in binge eating. Moreover, the impact of gonadal hormones on binge eating may itself depend on the social context. Thus, advancing research on interactions between hormonal and psychosocial factors is vital to promoting a holistic understanding of sex differences in binge eating risk.
